# Use of Preventive Aspirin Among Older US Adults With and Without Diabetes

**DOI:** 10.1001/jamanetworkopen.2021.12210

**Published:** 2021-06-21

**Authors:** Elizabeth Y. Liu, Mohammed E. Al-Sofiani, Hsin-Chieh Yeh, Justin B. Echouffo-Tcheugui, Joshua J. Joseph, Rita R. Kalyani

**Affiliations:** 1 Division of Endocrinology, Diabetes & Metabolism, Johns Hopkins University, Baltimore, Maryland; 2 Division of Endocrinology, King Saud University College of Medicine, Riyadh, Saudi Arabia; 3 Division of General Internal Medicine, Johns Hopkins University, Baltimore, Maryland; 4 Department of Epidemiology, Johns Hopkins University Bloomberg School of Public Health, Baltimore, Maryland; 5 Welch Center for Prevention, Epidemiology, and Clinical Research, Johns Hopkins University, Baltimore, Maryland; 6 Division of Endocrinology, Diabetes and Metabolism, The Ohio State University, Columbus; 7 Center on Aging and Health, Johns Hopkins University, Baltimore, Maryland

## Abstract

**Importance:**

The net benefit of aspirin for prevention of cardiovascular disease (CVD), particularly primary prevention, remains debated in people with and without diabetes. Recent studies suggest that the benefits of preventive aspirin may be outweighed by the potential for harm in older adults; therefore, it is important to monitor current aspirin use in order to minimize risk for future harm in the oldest segment of the population.

**Objective:**

To determine the prevalence of preventive aspirin use in older US adults with and without diabetes for both primary and secondary prevention by age, sex, and CVD risk category.

**Design, Setting, and Participants:**

This cross-sectional analysis used nationally representative data from the National Health and Nutrition Examination Survey from 2011 to 2018. A total of 7103 individuals 60 years or older with and without diabetes completed a questionnaire on preventive aspirin use. Statistical analyses were performed from July 1, 2019, to April 1, 2021.

**Main Outcomes and Measures:**

Preventive aspirin use was defined as participants’ self-reported use of low-dose aspirin therapy based on their physician’s advice or their own decision.

**Results:**

A total of 7103 individuals (mean [SD] age, 69.6 [0.1] years; 45.2% men; 75.8% White participants) were evaluated. Overall, 61.7% of older US adults with diabetes vs 42.2% without diabetes used aspirin. Among people with diabetes, in multivariable logistic models adjusting for race, sex, education, CVD risk category, and body mass index, the likelihood of aspirin use in older vs younger age categories (reference: 60-69 years) did not differ. Among people without diabetes, aspirin use was significantly greater in older age categories vs the reference (model 3, 70-79 years, odds ratio [OR], 1.50; 95% CI, 1.23-1.83; model 3, ≥80 years, OR, 1.59; 95% CI, 1.24-2.04). An estimated 9.9 million US adults 70 years or older with or without diabetes reported taking aspirin for primary prevention. The likelihood of aspirin use for primary prevention in those at high vs low risk for CVD did not differ among older adults with diabetes (model 3, OR, 1.69; 95% CI, 0.65-4.39) but was significantly higher in those without diabetes (model 3, OR, 2.46; 95% CI, 1.63-3.71). Women vs men with diabetes were less likely to be using aspirin for primary prevention (model 3, OR, 0.63; 95% CI, 0.48-0.83).

**Conclusions and Relevance:**

This cross-sectional study found that preventive aspirin use was higher among older adults with diabetes than in those without diabetes. Results suggest that 9.9 million older US adults who previously took aspirin for primary prevention would not be recommended for its continued use, particularly among those with diabetes.

## Key Points

**Question:**

What is the prevalence of aspirin use among older adults with and without diabetes in the US?

**Findings:**

This cross-sectional study of US adults from 2011 to 2018 found that 61.7% of adults 60 years and older with diabetes vs 42.2% of adults without diabetes used aspirin. Use of aspirin increased with older age and higher cardiovascular disease risk among those without diabetes, whereas it was uniformly high among those with diabetes.

**Meaning:**

In the context of recently revised guidelines that discourage routine use of aspirin in adults 70 years and older, these findings suggest that older adults may have potential overuse of aspirin therapy if it is not actively discontinued, especially among those with diabetes.

## Teaser

This cross-sectional study uses data from the National Health and Nutrition Examination Survey to assess the prevalence of aspirin use among older adults in the US with and without diabetes.

## Introduction

Aspirin is one of the world’s oldest and most used medications,^1^ and the role of aspirin in preventing cardiovascular events in people with a history of cardiovascular disease (CVD) has been well established.^1,2^ However, the net benefit of aspirin use for primary prevention of CVD remains debated in people with and without diabetes. Although the risk of developing CVD can be 2 to 4 times higher in individuals with diabetes than in individuals without diabetes,^3^ there is less clarity regarding the role of aspirin in primary prevention for both of these populations.

In 2015, the American Heart Association and the American Diabetes Association (ADA) released a joint statement recommending that aspirin use for primary prevention of CVD in individuals with diabetes is “…reasonable among those at 10-year CVD risk of at least 10% without increased risk of bleeding…” and “…in adults with diabetes at intermediate risk (10-year CVD risk, 5%-10%).”^4^^(p1786)^ In 2016, the US Preventive Services Task Force recommended that individual judgment should be used when considering preventive aspirin use for adults in the general population aged 60 to 69 years with a 10-year CVD risk of 10% or greater; no definitive recommendation was given for adults 70 years or older.^5^ The revised 2018 ADA guidelines continued to recommend aspirin for primary prevention in individuals with diabetes who have an increased CVD risk and are not at an increased risk of bleeding (men and women 50 years or older with at least 1 additional major risk factor for CVD).^6^ The ADA did not recommend aspirin use for those at low risk of CVD.^6^

In 2018, data from 3 randomized clinical trials found a significantly increased risk of adverse bleeding events with variable benefits associated with aspirin use for primary prevention of CVD among older adults with and without diabetes.^7,8,9,10^ The ARRIVE (Aspirin to Reduce Risk of Initial Vascular Events) trial found no difference in cardiovascular events when comparing aspirin use to placebo in an average-risk population 55 years (men) or 60 years (women) or older without diabetes.^8^ The ASPREE (Aspirin in Reducing Events in the Elderly) trial found that aspirin use for primary prevention in the general population of adults 70 years or older or in Black and Hispanic individuals 65 years or older (11% with diabetes) showed no benefit in CVD prevention compared with placebo for adults without CVD, dementia, or disability^9^ but significantly increased the risk of hemorrhage and all-cause mortality.^9,10^ Importantly, the ASCEND (A Study of Cardiovascular Events in Diabetes) trial found that aspirin use for primary prevention among individuals with diabetes 40 years or older significantly lowered the risk of serious vascular events; however, the incidence of major bleeding was higher as well, although subgroup analysis did not suggest significant differences by age.^7^ Given these findings, use of aspirin for primary prevention demands a patient-centered approach to evaluate risks and benefits.

In response, several professional societies have updated their guidelines on preventive aspirin use and called for careful consideration in older adults.^11,12,13^ With these recent paradigm shifts, it is important to understand the current landscape of aspirin use for CVD prevention in order to appropriately inform clinical management of populations most at risk for adverse events. The extent to which older populations with vs without diabetes currently use aspirin for CVD prevention, as well as the demographic and clinical factors associated with preventive aspirin use, have, to our knowledge, not been thoroughly studied. In this study, we aimed to characterize the prevalence of preventive aspirin use by CVD status in a nationally representative sample of US adults with and without diabetes who were 60 years or older from 2011 to 2018 and to investigate the presence of potential overuse of preventive aspirin therapy in older adults.

## Methods

This cross-sectional study used data from the National Health and Nutrition Examination Survey (NHANES), which is a large, cross-sectional survey conducted by the National Center for Health Statistics. The NHANES is designed to provide nationally representative estimates regarding health and disease in the US civilian community-dwelling population. Details regarding data collection methods can be found on the NHANES website.^14^

Our study combines data from the 2011 to 2018 cycles. Use of aspirin did not significantly differ over time; thus, pooled results are presented.^15^ There were 39 156 total participants, with 37 399 who participated in both the interview and physical examination. Of these, 7299 participants were 60 years or older. Those who had missing information on diabetes status (n = 4) and/or missing information on CVD status (n = 184) were excluded from the study. Those who did not indicate whether they were using aspirin (n = 8) were also excluded from this study. Our final sample consisted of 7103 adults 60 years or older. The research ethics boards of the National Center for Health Statistics approved NHANES. All participants provided written informed consent. Informed consent was not sought again for this analysis in accordance with 45 CFR §46. Our report followed the Strengthening the Reporting of Observational Studies in Epidemiology (STROBE) reporting guideline.

### Assessment of Diabetes Status

Individuals were classified as having diabetes if they self-reported (1) a physician diagnosis of diabetes and/or (2) use of a glucose-lowering medication. We were interested in comparing those with known diabetes to those without diabetes, as this could affect aspirin prescribing practices; laboratory measures were not used in the definition. Data regarding duration of diabetes were self-reported via questionnaire.

### Assessment of Current Aspirin Use

Data on current aspirin use as well as physician-recommended aspirin use were ascertained from the “Preventive Aspirin Use” questionnaire, which was administered by trained interviewers to all survey participants 40 years and older between 2011 and 2018. We classified participants as using preventive aspirin if they indicated that they (1) followed their physician’s advice and took low-dose aspirin (including sometimes) or (2) were taking low-dose aspirin on their own to prevent heart attacks, stroke, or cancer. Schedule, frequency, and aspirin dose data were not uniformly available and thus not included in analysis.

### Assessment of Demographic, Clinical, and Laboratory Measures

Data on age, sex, race/ethnicity, education level, and smoking status were ascertained from the demographic variables of the NHANES survey. Body mass index (BMI) (calculated as weight in kilograms divided by height in meters squared) and waist circumference (WC) were measured during the physical examination. Obesity was defined as BMI greater than or equal to 30 kg/m^2^. Blood pressure was measured a maximum of 4 times during the physical examination. During the questionnaire portion, we classified participants as having hypertension if they self-reported use of antihypertensive medications or a physician diagnosis of hypertension, or if they had an average systolic blood pressure of at least 140 mm Hg and/or an average diastolic blood pressure of at least 90 mm Hg during the physical examination. Total cholesterol and high-density lipoprotein (HDL) were measured during the laboratory portion of the examination, regardless of fasting status. Non-HDL cholesterol was calculated by subtracting HDL cholesterol levels from total cholesterol levels. We classified participants as having hypercholesterolemia if they self-reported use of cholesterol-lowering medications or a physician diagnosis of hypercholesterolemia, or if their total cholesterol was at least 240 mg/dL, similar to that previously described.^16^ We classified participants as having albuminuria if their ratio of urine albumin to creatinine was 30 mg/g or greater during the laboratory portion of the examination.

Personal history of coronary heart disease, angina pectoris, stroke, congestive heart failure, and myocardial infarction (MI), as well as family history of MI (close biological relative with MI younger than 50 years), were self-reported during the questionnaire portion of the survey. Interviewers asked participants 40 years or older questions from the Cardiovascular Health/Rose Questionnaire, which were used to determine the presence of angina pectoris.^17^ Participants who self-reported a history of coronary heart disease, angina, stroke, or MI were considered to have CVD. Risk factors for CVD included obesity, hypertension, albuminuria, hyperlipidemia, smoking history (current or recent smoker who quit less than 5 years ago),^18^ or family history of MI. Hemoglobin A_1c_ measurements were performed as previously described.^16^

### Statistical Analysis

All analyses were performed using Stata/IC software, version 15.1 (StataCorp) and incorporated population-based sampling weights to obtain unbiased, nationally representative estimates from the complex NHANES sampling design. The standard errors of the means for all estimates were obtained using Taylor linearization following the National Center for Health Statistics–recommended procedures.^19^ The Wald test was used to compare differences in demographic and clinical characteristics by current aspirin use status for older adults categorized as follows: with a history of CVD, without a history of CVD but with diabetes, and without a history of CVD or diabetes. Logistic regression models were used to assess the association of diabetes status with aspirin use in both the overall population and primary prevention subset. Model 1 was unadjusted; model 2 was adjusted for age, race/ethnicity, sex, education, and CVD risk status; and model 3 was adjusted for model 2 plus BMI.

In a sensitivity analysis, we performed additional logistic regression analyses to examine the relationship between older age and physician-prescribed low-dose aspirin by diabetes and CVD status. We also performed sensitivity analyses by replacing BMI with WC as well as using non-HDL cholesterol in our regression models. A 2-tailed *P* < .05 was considered significant. Statistical analyses were performed from July 1, 2019, to April 1, 2021.

## Results

A total of 7103 individuals (mean [SD] age, 69.6 [0.1] years; 45.2% men; 75.8% White participants) were evaluated. The prevalence of aspirin use in older US adults was 46.7% overall; 61.7% among people with diabetes vs 42.2% among those without a history of diabetes. The prevalence of diabetes in US adults 60 years or older was 23.0% (95% CI, 21.6%-24.3%). An estimated 9.9 million US adults 70 years or older with or without diabetes reported use of aspirin therapy for primary prevention.

Among individuals without a history of CVD but with diabetes, those who took aspirin were also more likely to be men (54.0%; SE, 2.5%) and have a higher mean WC (mean [SD], 110.2 [0.8] cm) (Table 1). There were no significant differences regarding mean age, BMI, race/ethnicity, or hemoglobin A_1c_. Among adults without a history of CVD or diabetes, aspirin users had a higher mean (SD) age (aspirin use, 70.2 [0.3] years vs no aspirin use, 68.4 [0.2] years) and were more likely to be White (aspirin use, 82.7% [SE, 1.5%] vs no aspirin use, 76.4% [SE, 1.6%]) compared with those not using aspirin. They also had a higher mean (SD) WC (102.1 [0.6] cm vs 99.8 [0.5] cm), BMI (28.9 [0.3] kg/m^2^ vs 28.2 [0.2] kg/m^2^), and hemoglobin A_1c_ (5.67% [0.01%] vs 5.62% [0.01%]) and were more likely to have hypertension (61.4% [SE, 2.1%] vs 42.9% [SE, 1.6%]) or hypercholesterolemia (68.8% [SE, 1.6%] vs 53.4% [SE, 1.2%]). Among individuals with a history of CVD (38.0% with diabetes), aspirin users were more likely to be men (aspirin, 61.1% [SE, 2.5%] vs no aspirin, 47.8% [SE, 2.9%]) (Table 1).

**Table 1.** Prevalence of Preventive Aspirin Use by Cardiovascular Disease and Diabetes Status According to Demographic and Clinical Characteristics Among 7103 US Adults Aged 60 Years or Older, NHANES 2011-2018^a^

Characteristic	History of CVD (n = 1496)^b^			No history of CVD						
				Diabetes (n = 1378)				No diabetes (n = 4229)		
	No ASA	ASA	*P* value	No ASA	ASA	*P* value	No ASA		ASA	*P* value
Age group, y										
60-69	37.0 (2.9)	40.1 (2.1)	.047	56.6 (2.8)	53.8 (2.4)	.61	62.1 (1.5)		50.3 (2.2)	<.001
70-79	31.6 (2.8)	36.3 (1.7)		30.6 (2.5)	33.6 (2.6)		25.7 (1.3)		31.4 (1.5)	
≥80	31.4 (2.3)	23.6 (1.6)		12.8 (1.3)	12.6 (1.5)		12.1 (0.8)		18.3 (1.5)	
Mean (SD) age, y	72.2 (0.4)	71.6 (0.3)	.23	69.1 (0.4)	69.5 (0.3)	.41	68.4 (0.2)		70.2 (0.3)	<.001
Sex										
Male	47.8 (2.9)	61.1 (2.5)	<.001	43.4 (2.9)	54.0 (2.5)	.007	40.3 (1.3)		40.7 (1.6)	.85
Female	52.2 (2.9)	38.9 (2.5)	<.001	56.6 (2.9)	46.0 (2.5)	.007	59.7 (1.3)		59.3 (1.6)	.85
Race/ethnicity										
White	73.6 (2.4)	78.3 (1.8)	.27	58.7 (3.4)	64.7 (2.8)	.17	76.4 (1.6)		82.7 (1.5)	<.001
Black	9.5 (1.3)	8.3 (1.0)		14.7 (1.9)	13.0 (1.8)		8.3 (0.9)		7.8 (0.9)	
Mexican American	3.6 (0.9)	2.9 (0.7)		9.7 (2.1)	7.1 (1.2)		4.2 (0.6)		2.6 (0.5)	
Asian	2.8 (0.7)	3.0 (0.4)		8.2 (1.5)	6.4 (1.0)		5.3 (0.6)		2.0 (0.3)	
Other	10.5 (1.9)	7.5 (1.3)		8.6 (1.3)	8.9 (1.5)		5.8 (0.6)		4.8 (0.7)	
Education level										
<High school	23.9 (2.1)	19.5 (1.9)	.05	24.7 (2.3)	18.7 (1.8)	.07	14.4 (1.1)		12.6 (1.2)	.33
High school	30.4 (3.0)	25.5 (1.9)		20.6 (2.2)	27.0 (2.9)		22.8 (1.3)		24.3 (1.8)	
>High school	45.6 (3.0)	55.0 (2.2)		54.7 (2.9)	54.3 (2.6)		62.7 (1.5)		63.2 (2.1)	
Smoking										
Current	16.2 (2.4)	14.0 (1.8)	.75	9.3 (2.0)	8.2 (1.4)	.56	12.4 (0.9)		8.7 (1.1)	.06
Former	44.3 (3.5)	44.7 (2.5)		38.6 (2.8)	42.8 (2.6)		34.3 (1.5)		37.3 (2.1)	
Never	39.5 (3.0)	41.3 (2.4)		52.1 (2.4)	49.0 (2.8)		53.3 (1.4)		54.1 (2.0)	
Metabolic status, mean (SD)										
BMI	30.2 (0.5)	29.8 (0.3)	.54	31.4 (0.4)	32.1 (0.4)	.25	28.2 (0.2)		28.9 (0.3)	.04
Waist circumference, cm	106.1 (1.3)	106.4 (0.9)	.84	107.6 (0.8)	110.2 (0.8)	.04	99.8 (0.5)		102.1 (0.6)	.002
HbA_1c_, %	6.31 (0.09)	6.20 (0.05)	.28	7.06 (0.09)	7.07 (0.08)	.95	5.62 (0.01)		5.67 (0.01)	.01
Diabetes duration, y	NA	NA	NA	12.5 (0.6)	13.9 (0.6)	.16	NA		NA	NA
Comorbidities										
Coronary heart disease	63.8 (2.9)	80.6 (1.8)	<.001	NA	NA	NA	NA		NA	NA
Stroke	52.6 (3.3)	31.4 (2.2)	<.001	NA	NA	NA	NA		NA	NA
Congestive heart failure	24.6 (2.1)	23.6 (1.7)	.72	5.7 (1.4)	4.0 (0.9)	.30	1.5 (0.3)		2.3 (0.4)	.11
Myocardial Infarction	38.7 (2.7)	48.1 (2.1)	.009	NA	NA	NA	NA		NA	NA
Angina	4.4 (1.1)	3.8 (0.9)	.70	NA	NA	NA	NA		NA	NA
Obesity	45.3 (3.4)	44.5 (2.8)	.85	51.1 (2.8)	57.9 (2.8)	.11	32.7 (1.5)		36.7 (1.8)	.06
Hypertension	72.3 (2.8)	77.3 (2.0)	.15	68.5 (3.6)	76.3 (2.0)	.06	42.9 (1.6)		61.4 (2.1)	<.001
Hypercholesterolemia	76.9 (2.5)	84.6 (1.5)	.008	72.9 (2.5)	77.8 (2.4)	.19	53.4 (1.2)		68.8 (1.6)	<.001
Family history of MI prior to age 50	23.9 (3.1)	22.9 (1.9)	.78	12.8 (2.2)	15.1 (2.1)	.45	11.4 (0.8)		13.9 (1.4)	.10

Abbreviations: BMI, body mass index (calculated as weight in kilograms divided by height in meters squared); CVD, cardiovascular disease; HbA_1c_, hemoglobin A_1c_; MI, myocardial infarction; NA, not available; NHANES, National Health and Nutrition Examination Survey.

^a^ Nationally representative estimates shown as % (linearized SE), unless indicated otherwise, percentages within columns are shown.

^b^ Includes adults both with or without diabetes. History of CVD includes myocardial infarction, stroke, angina, and coronary heart disease.

Within age group strata (Figure), the prevalence of aspirin use among those with vs without diabetes was significantly higher in those aged 60 to 69 years and 70 to 79 years (60-69 years, 62.2% [2.4%] vs 36.0% [1.8%] and 70-79 years, 61.9% [2.4%] vs 48.5% [1.6%]; both *P* < .001). The prevalence of aspirin use in the group 80 years or older was similar in those with vs without diabetes (59.1% [3.3%] vs 51.8% [1.9%]; *P* = .06).

**Figure.**
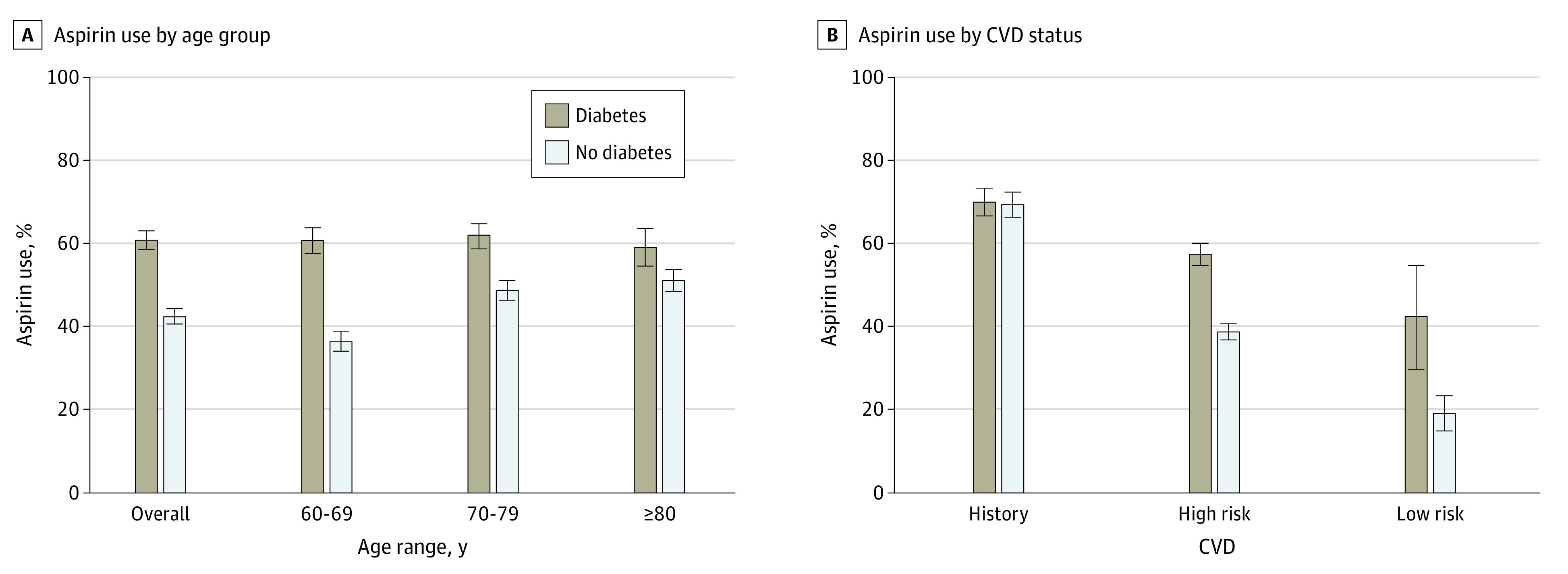
Aspirin Use by Age Group and Cardiovascular Disease (CVD) Status in Older Adults With (n = 1987) vs Without (n = 5116) Diabetes

When stratifying by CVD status (Figure), the prevalence of aspirin use was significantly higher in those with vs without diabetes for those at higher risk (ie, with 1 or more CVD risk factors but no history of CVD; 57.8% [2.0%] vs 39.3% [1.2%]; *P* < .001) as well as for those at low risk (ie, with no history of CVD and no risk factors; 43.0% [12.0%] vs 19.9% [3.3%]; *P* = .02). There was no significant difference in prevalence of aspirin use for those with a history of CVD with or without diabetes (70.3% [2.5%] vs 69.8% [2.2%]; *P* = .88).

In logistic regression models, diabetes was associated with significantly higher odds of aspirin use after adjusting for age, race/ethnicity, sex, education, CVD risk, and BMI in the overall study population (odds ratio [OR], 1.98; 95% CI, 1.47-2.67). The likelihood of aspirin use for primary or secondary prevention in those at high vs low risk for CVD did not differ among older adults with diabetes (model 3, OR, 1.69; 95% CI, 0.65-4.39). Among older adults with diabetes, a history of CVD (vs low risk for CVD status) was associated with a significantly higher odds of aspirin use (model 3, OR, 3.00; 95% CI, 1.18-7.59), and female (vs male) sex was associated with a significantly lower odds of aspirin use after adjusting for covariates (model 3, OR, 0.63; 95% CI, 0.48-0.83) (Table 2). Among those without diabetes, older (vs younger) age (model 3, 70-79 years, OR, 1.50; 95% CI, 1.23-1.83; model 3, ≥80 years, OR, 1.59; 95% CI, 1.24-2.04) and high risk for CVD status (model 3, OR, 2.46; 95% CI, 1.63-3.71) or history of CVD (model 3, OR, 8.27; 95% CI, 5.54-12.34) (vs low risk for CVD) were associated with a significantly higher odds of aspirin use after adjusting for covariates. Female sex was found to be associated with a significantly lower odds of aspirin use in model 1 among those without diabetes (OR, 0.81; 95% CI, 0.70-0.95), but the relationship became nonsignificant in adjusted models.

**Table 2.** Logistic Regression Models for the Association of Age, Sex, and Cardiovascular Disease History With Aspirin Use for Primary or Secondary Prevention in 7103 US Adults Aged 60 Years or Older With and Without Diabetes, NHANES 2011-2018

Variable	History of diabetes, OR (95% CI)			No history of diabetes, OR (95% CI)		
	Model 1^a^	Model 2	Model 3	Model 1	Model 2	Model 3
Age category, y						
60-69	1 [Reference]	1 [Reference]	1 [Reference]	1 [Reference]	1 [Reference]	1 [Reference]
70-79	0.99 (0.74-1.31)	0.95 (0.72-1.26)	0.95 (0.72-1.26)	1.67 (1.39-2.02)	1.50 (1.23-1.83)	1.50 (1.23-1.83)
≥80	0.88 (0.63-1.23)	0.84 (0.59-1.20)	0.87 (0.61-1.26)	1.91 (1.53-2.40)	1.54 (1.20-1.98)	1.59 (1.24-2.04)
*P* value for trends	.58	.76	.98	<.001	<.001	<.001
Sex						
Male	1 [Reference]	1 [Reference]	1 [Reference]	1 [Reference]	1 [Reference]	1 [Reference]
Female	0.61 (0.46-0.79)	0.63 (0.49-0.82)	0.63 (0.48-0.83)	0.81 (0.70-0.95)	0.87 (0.74-1.03)	0.89 (0.75-1.05)
CVD history^b^						
Low risk	1 [Reference]	1 [Reference]	1 [Reference]	1 [Reference]	1 [Reference]	1 [Reference]
High risk	1.82 (0.67-4.96)	1.76 (0.68-4.59)	1.69 (0.65-4.39)	2.60 (1.73-3.93)	2.51 (1.66-3.79)	2.46 (1.63-3.71)
History of CVD	3.14 (1.17-8.43)	2.93 (1.14-7.49)	3.00 (1.18-7.59)	9.27 (6.08-14.12)	8.23 (5.41-12.53)	8.27 (5.54-12.34)

Abbreviations: CVD, cardiovascular disease; MI, myocardial infarction; NHANES, National Health and Nutrition Examination Survey; OR, odds ratio.

^a^ Model 1, unadjusted; Model 2, adjusted for age, sex, race/ethnicity, education, CVD risk status (all covariates included except the variable used to stratify participants in each of the respective models above); Model 3, Model 2 plus body mass index.

^b^ Low risk, no CVD risk factors; high risk, 1 or more CVD risk factors. CVD risk factors include family history of MI, obesity, hypertension, smoking, albuminuria, and hypercholesterolemia.

The association of older age with aspirin use for primary prevention (ie, among those without prior history of CVD) is shown in Table 3. In adults with diabetes, older age was not significantly associated with aspirin use. However, in adults with no history of diabetes, older age was significantly associated with greater likelihood of aspirin use compared with the reference (model 1, age ≥80 years, OR, 1.86; 95% CI, 1.43-2.42). When we adjusted for race/ethnicity, sex, education, and BMI, we found that those in the age group of 70 to 79 years with no history of diabetes had a significantly higher odds of aspirin use for prevention (model 3, OR, 1.54; 95% CI, 1.23-1.92), and those in the group 80 years or older had approximately 2-fold higher odds of aspirin use for primary prevention compared to the reference (model 3, OR, 1.96; 95% CI, 1.48-2.58; *P* value for trend < .001). In sensitivity analyses, we found similar results when including physician-prescribed aspirin use, WC, and non-HDL cholesterol levels in regression models.

**Table 3.** Logistic Regression Models for the Association of Age With Aspirin Use for Primary Prevention Among 5607 US Adults Aged 60 Years or Older by Diabetes Status, NHANES 2011-2018

Variable	OR (95% CI)		
	Model 1^a^	Model 2	Model 3
**History of diabetes (n = 1378)**			
Age	1.01 (0.99-1.03)	1.01 (0.99-1.03)	1.01 (0.99-1.04)
Age categories, y			
60-69	1 [Reference]	1 [Reference]	1 [Reference]
70-79	1.16 (0.82-1.63)	1.15 (0.82-1.60)	1.13 (0.80-1.59)
≥80	1.04 (0.72-1.49)	1.07 (0.74-1.57)	1.15 (0.77-1.72)
*P* value for trends	.55	.66	.38
**No history of diabetes (n = 4229)**			
Age	1.04 (1.03-1.06)	1.04 (1.03-1.06)	1.04 (1.03-1.06)
Age categories, y			
60-69	1 [Reference]	1 [Reference]	1 [Reference]
70-79	1.51 (1.21-1.87)	1.51 (1.21-1.89)	1.54 (1.23-1.92)
≥80	1.86 (1.43-2.42)	1.84 (1.41-2.40)	1.96 (1.48-2.58)
*P* value for trends	<.001	<.001	<.001

Abbreviations: NHANES, National Health and Nutrition Examination Survey; OR, odds ratio.

^a^ Model 1, unadjusted; Model 2, adjusted for sex, race/ethnicity, education; Model 3, Model 2 plus body mass index.

## Discussion

We estimated that half of older US adults report preventive aspirin use. Our study results suggest that people with diabetes are twice as likely to be using aspirin for primary prevention than those without diabetes. Women were significantly less likely to be using aspirin than men, particularly among those with diabetes. Greater age was associated with a higher likelihood of aspirin use in those without diabetes but not in those with diabetes, who had a uniformly high prevalence of aspirin use above the age of 60 years. Findings were similar among older adults who used aspirin for primary prevention.

Compared with studies using data from the late 1980s to mid-1990s^20^ and early 2000s,^21^ we found an increase in the prevalence of aspirin use in populations with diabetes. A report using data from NHANES III 1988-1994 found that the prevalence of aspirin use in those 60 years or older with diabetes was 25%, and only 37% of US adults aged 21 years or older with diabetes and a history of CVD used aspirin at that time.^20^ A telephone survey study from 1997 to 2001 found that in people 65 years or older with diabetes, 74.0% with CVD used aspirin, and 46.7% without CVD used aspirin.^21^ In contrast, our study found much higher numbers of older adults without CVD used aspirin for primary prevention than reported previously. Our findings were consistent with a recent report that analyzed 1-year data from the 2017 National Health Interview Survey, which found that more than 40% of adults older than 70 years in the general population without CVD reported taking aspirin.^22^ Our study complements and extends the findings from prior studies by further investigating the use of aspirin in older US adults with and without diabetes from 2011 to 2018 and identifies the presence of potential overuse of aspirin preventive therapy if not actively reconsidered by their physicians or discontinued given current clinical guidelines.

The increase in the prevalence of aspirin use in older populations with diabetes over recent decades may be due to a multitude of factors. After the National Cholesterol Education Program guidelines were published in 2001,^23^ public health efforts were initiated to promote aspirin use in adults with diabetes (“Be Smart About Your Heart” campaign from the National Institutes of Health and “Make the Link!” campaign from the ADA/American College of Cardiology).^21^ However, as guidelines narrowed the eligible population over the years, older age was still not considered a risk factor for adverse effects from aspirin use when considering primary prevention in individuals with or without diabetes. In fact, before 2019, many medical societies recommended aspirin use in those older than 50 years without an upper cutoff age limit.^4,5,6,22^ In 2019, the ADA updated their guidelines, calling for careful consideration and generally discouraging aspirin use for primary prevention in adults older than 70 years; however, aspirin could be considered for patients with high CVD risk and low bleeding risk, “but generally not in older adults.”^11pS.114^ In the same year, the American College of Cardiology and the American Heart Association released updated guidelines that recommended against routine aspirin use for primary prevention in adults older than 70 years.^13^ Our results suggest potential overuse of primary preventive aspirin therapy in approximately 9.9 million adults 70 years or older with or without diabetes if aspirin use is not actively discontinued. At the time of writing this article, the US Preventive Services Task Force was in the process of updating its recommendations.^24^

The sex differences in aspirin use for those with diabetes were noteworthy as well, especially in the context of previous studies that suggested that diabetes has a relatively greater effect on increasing CVD risk in women vs men.^25,26^ Our findings were consistent with previous studies that have found lower aspirin use in women among the general population as well as among adults with diabetes.^21,22^ Sex disparities in aspirin use could be due to several factors, including less awareness of CVD risk among women,^27^ misconceptions that women are protected against CVD,^25^ and concerns that aspirin is less effective for women than for men, an idea that has been refuted by recent studies.^28^ This disparity warrants further investigation in future studies.

### Strengths and Limitations

The strengths of our study included the ability to generate nationally representative estimates from the NHANES that were generalizable to the US population. The NHANES included a detailed aspirin questionnaire, which allowed us to ascertain aspirin use in a comprehensive manner among participants by diabetes and CVD status. Furthermore, our results included the most recently released data cycles, which rendered our findings timely and relevant to current clinical practice.

The limitations of this study included that some variables, such as aspirin use and diagnosis of diabetes, were based on self-reported data, which could have led to misclassification. We could not exclude the possibility that people who reported taking glucose-lowering medications may have included those with prediabetes who were misclassified into the diabetes group. However, this would potentially underestimate differences in aspirin use between groups, and we still observed significant findings. Furthermore, the NHANES preventive aspirin use questionnaire included those taking aspirin for cancer prevention as well and could not distinguish between those taking aspirin for CVD prevention or cancer prevention. We used a cross-sectional study design and cannot draw conclusions related to temporality of any observed associations.

## Conclusions

In this cross-sectional study, results suggest that the prevalence of aspirin use among older US adults is high among populations at an increased risk for harm, particularly older individuals and individuals with diabetes, who already are at an increased bleeding risk compared with those without diabetes.^29^ These findings suggest a greater need for health care providers to ask their older patients about aspirin use and, given recent guideline changes, to discuss the risks and benefits of continuing aspirin treatment for CVD prevention. Ultimately, future studies should examine the influence of updating guidelines on clinician behaviors and the association of changing trends in preventive aspirin use with the development of CVD in older adults.
